# Sociodemographic and cultural factors are related to singlehood rates: A multilevel analysis across 59 countries from the World Values Survey

**DOI:** 10.1371/journal.pone.0335416

**Published:** 2025-10-29

**Authors:** Marta Kowal, Katarzyna Adamczyk

**Affiliations:** 1 IDN Being Human Lab – Institute of Psychology, University of Wrocław, Wrocław, Poland; 2 Faculty of Psychology and Cognitive Science, Adam Mickiewicz University, Poznań, Poland; John Paul II Catholic University of Lublin: Katolicki Uniwersytet Lubelski Jana Pawla II, POLAND

## Abstract

Singlehood, which refers to remaining without a lifetime partner, has become an increasingly common phenomenon. However, there is still limited understanding of the individual-level sociodemographic and country-level cultural factors that predict one’s singlehood status. We addressed this question by utilizing data from the World Values Survey, which included responses from 71,169 individuals across 59 countries. Through multilevel modeling, we discovered that several factors increase the likelihood of being single. These factors include being younger, being male, residing in a larger town, having a higher level of education, having a lower income, being unemployed, and living in countries characterized by higher individualism and lower flexibility. Additionally, the likelihood of being single varied according to country-level individualism and flexibility, interacting with various individual-level factors. These findings suggest that the significance of individual sociodemographic characteristics on the prevalence of single individuals depends on country-level traits related to individualism-collectivism and flexibility-monumentalism.

## Introduction

In recent decades, we have observed significant changes in family forms and household structures in many countries [1]. These transformations also involve the rise of a single population in the United States of America and Canada, as well as in many European and Asian countries [2,3]. The increasing shares of single individuals intensify further scientific pursuits of determinants of singlehood prevalence and associated life outcomes [4,5].

Numerous studies have explored the determinants associated with remaining single, ranging from sociodemographic to psychological factors [3,4,6,7]. For instance, 1) a shift in values associated with the second demographic transition relates to a move toward stronger individualistic values, lower dependence on the family, and increasing attempts at self-realization [3,7]. 2) The contraceptive and sexual revolution allows for the postponement of relationship establishment and childbearing and, in connection with the higher acceptance and prevalence of sex outside of marriage and premarital sex, enables people to remain single for longer periods [3]. 3) Educational expansion delays the age at which people complete their education and causes a delay in labor market entry that contributes to the postponement of union formation [3,8]. 4) The increasing uncertainty in the labor market, lower stability in the economic situation, expensive housing, and rising levels of debt [3,9,10] have all been related to the postponement of marriage, particularly among men [9]. In other words, a lack of economic well-being and stability may discourage people from establishing serious relationships [3,9,10]. 5) Social multiplier effects [11] relate to the observation that other people live alone and that there are greater opportunities, infrastructure, and networks for single individuals, encouraging them to live as singles [3]. Urban residency has been associated with reduced adherence to traditional values [9,12], thereby creating an environment that fosters greater acceptance of non-traditional marital and family structures such as singlehood as a less traditional life path [4,7,12]. On the other hand, individuals living in more compact urban places have been found to have greater opportunities to meet new people than individuals living in low-density suburban neighborhoods [13], which might suggest that compact urban residences increase the opportunities for people to find a partner.

Age and sex were also factors analyzed in the context of singlehood. For instance, age has often been used in defining who is a single person. For example, Kaiser and Kashy [14] utilized the age of 36.2 years to distinguish normative from nonnormative singles, with individuals above this age treated as nonnormative singles. In turn, Kislev [12] considered singlehood only among people who were older than 30 years since the age of 30 was the mean age at first marriage in European countries, as well as the age at which people face the intensification of internal and external expectations to marry and the consequences of remaining single. At the same time, in the period of mid to late adulthood (approximately 40s–80s), individuals become more satisfied with their single status [15], and this tendency can cause people to remain single.

With respect to sex, past literature has suggested that women and men may experience different degrees of singlehood [4]. In general, the past literature has suggested that women are more relational than men are and desire more social connectedness. In turn, men are considered to be more oriented toward activities than toward being in relationships [16]. At the same time, women are considered to encounter greater social pressure to find a partner and cohabitate or marry [17]. Indeed, previous studies have suggested that single women may experience more negative perceptions than single men due to societal expectations [4].

While the sociodemographic factors related to singlehood have been well explored [2,3,7], less is known about whether sociodemographic factors operate in a similar way with regard to the cultural traits measured at the country level. The limited number of studies in this field is noteworthy, particularly in light of previous findings that indicate cultural values significantly shape individuals’ beliefs and behaviors [18]. Furthermore, developmental tasks, such as forming an intimate partnership, are influenced by sociocultural constructs that mirror the evolving historical and cultural contexts of human development [19]. Cultural norms are pivotal in defining social and intimate relationships, as they clarify which connections provide essential social support and identify attributes that individuals consider important in their lives [20]. Additionally, although loneliness should not be equated with singlehood, it is a phenomenon that is shaped by cultural factors [20].

Hofstede’s classic model of national culture has had a significant impact on the field of cultural studies [21,22], despite the identification of many cultural dimensions in the literature. A substantial number of studies examining people’s behaviors—particularly regarding social relationships and loneliness—have focused on Hofstede’s model [20,23,24]. Furthermore, a recent study exploring the link between relationship status (single vs. partnered) and loneliness during the COVID-19 pandemic analyzed the moderating role of cultural values based on Hofstede’s cultural dimensions [25].

However, while Hofstede’s model has gained popularity, it has never been fully replicated. Its predictive properties have faced challenges, leading to revised models [22]. For example, Minkov [22] revised Hofstede’s model, reducing it to two dimensions: Individualism-Collectivism (IDV-COLL) and a Long-term Orientation, which he renamed Flexibility-Monumentalism (FLX-MON). The Minkov-Hofstede model reflects subjective culture in relation to objective culture, incorporating aggregated measures of various behaviors, such as transparency and corruption, political freedom, gender equality, road death tolls, educational achievement, homicide rates, adolescent fertility, and family structure (e.g., paternal presence at home versus absenteeism) [26]. This revised model has proven effective in replicating results across different countries, exhibiting high internal reliability and predictive power regarding critical external variables, thereby establishing a strong foundation for cross-cultural studies [26].

The individualism dimension possesses three distinctive features: 1) freedom to behave in line with one’s own beliefs and decide which societal rules to abide, particularly in the domains of religion, sexuality, the creation and termination of life, and traditional gender roles; 2) assertiveness and conflict acceptance; and 3) universalism, which reflects the conviction that all individuals have some universal rights [26]. In turn, collectivism is distinguished by the following three features: 1) religious conservatism, conformism, and importance of tradition, which are related to the strict obedience of societal rules, including restrictive sexual morality and separation of gender roles; 2) submissiveness and conflict avoidance; and 3) exclusionism, which results in exclusion and discrimination (including ethnocentrism, nepotism, sexism, and homophobia) of individuals who do not belong to a powerful in-group [26]. Furthermore, among culture-related practices, the following practices have been found to be strongly associated with the IDV-COLL rule of law (including transparency vs. corruption), political freedom, social (in)equality, especially gender (in)equality, and fatal accident rates in transportation and industrial settings [22].

The second dimension, flexibility–monumentalism (FLX-MON), is an improved variant of Hofstede’s long-term orientation (LTO) vs. short-term orientation (STO) [27]. The flexibility pole, often exemplified in East Asian societies, is related to the reinforcement of adaptability to changing circumstances [27], self-sufficiency, self-enhancement, and the need for personal development, especially through education [26]. FLX reinforces notions of the self as flexible and humble [26], highlights self-control and suppression of desire for immediate gratification and, as a result, prompts a slow life-history strategy (LHS) [27]; moreover, both FLX and a slow LHS are considered to be prerequisites for long-term goals, such as obtaining a good education [27]. Flexible societies are characterized by high educational achievement, low violent crime rates, low levels of adolescent fertility, and low levels of paternal absenteeism [26].

In turn, at the monumentalism pole (e.g., Latin America, Africa, Arab world), the desired self is like a perennial and proud monument [26], and monumentalist cultures adhere to the belief that one should “always be the same: feel good about yourself, and make others feel good about you” [27]. In monumentalist societies, children are encouraged to follow impulses and desires and vent rather than control their feelings [26]. Such cultures also create networks of economically interdependent individuals; people are expected to be invariant, adhere to immutable values, be reliable and generous toward others and offer help to others [26].

Drawing from the notion that “culture has always been important for demography”

and “demographers need culture (…)” [28] and considering that Minkov’s revised two-dimensional variant of Hofstede’s subjective-culture model has not yet been assessed in tandem with sociodemographic characteristics as factors potentially related to the rates of single adults, the current study aimed to bridge the gaps in the existing research by linking individual sociodemographic characteristics (age, sex, size of town, education, income, employment) and cultural factors measured in terms of Minkov-Hofstede individualism–collectivism and flexibility–monumentalism to the rates of singlehood. Notably, This initial application of the Minkov-Hofstede model in the context of singlehood may serve to elucidate the observed differences in single adults rates across countries [29].

### The current study

Regarding the exploratory nature of the current analyses, in the present paper, we formulated one major, broad research question without specifying hypotheses: How do sociodemographic characteristics (age, sex, size of town, education, income, employment) measured at the individual level relate to the proportions of single adults across countries depending on Minkov-Hofstede dimensions of subjective culture (i.e., individualism–collectivism and flexibility–monumentalism)? To answer this question, we analyzed the World Values Survey [30] dataset in relation to the revised Minkov-Hofstede model of culture [22, 26,27].

At the same time, based on the traits of individualism–collectivism and flexibility–monumentalism and their associations with other culture-related practices [26,22], we may formulate general expectations concerning the associations between the cultural dimensions and the probability of remaining single. For instance, individualism is related, among others, to the freedom to behave in line with one’s own beliefs and decide which societal rules to abide by, particularly in the domains of religion, sexuality, the creation and termination of life, and traditional gender roles. Therefore, it might be expected that individuals in more individualistic societies may feel more freedom and approval when choosing a single life. Further, flexibility is associated with a more short-term approach to life, which may promote singlehood through numerous short-term relationships instead of a steady partner. On the other hand, flexibility is also associated with higher educational ambitions, which may delay union formation.

## Methods

### Participants and procedure

The World Values Survey received approval from the Institute for Comparative Survey Research Institutional Review Board. All participants were informed that their participation is voluntary and anonymous, and that their responses would only be used in a generalized format. In most countries, respondents provided explicit oral consent; in some countries, where legal requirements dictate or in the case of postal surveys, consent can be given in writing. The data for the current research were accessed on August 15, 2022. The authors of this study did not have access to any information that could identify individual participants during or after data collection.

The World Values Survey, Wave 7 (2017–2021) [30] dataset contains information from 93,701 participants from 64 countries. The data was collected through face-to-face interviews at the respondents’ home/residence. Respondents’ answers were recorded in a paper questionnaire or through Computer Assisted Personal Interviews. The samples were expected to be representative of all people aged 18 and older residing within private households in each country, regardless of their nationality, citizenship, or language [30].

In the current paper, we used data from a subsample of 81,374 individuals from 59 countries with no missing data on any of the variables of interest, including relationship status, age, sex, size of town, highest educational level attained, income, employment status, individualism–collectivism and flexibility–-monumentalism scores (i.e., individuals who had missing data on any of these variables were excluded from the analyses), among whom 71,169 were either “Single/Never married,” “Married,” or “Living as Married.” The detailed socio-demographic participants’ characteristics across the analyzed countries have been provided in S1 Table in S1 File.

## Measures

### Individual-level variables

***Relationship status variable*** (X007) was assessed with the question “Are you currently” with possible answers “married”, “living together as married”, “divorced”, “separated”, “widowed”, and “single/never married.”

***Age*** (X003) was assessed with the item “You are ____ years old.”

***Sex*** (X001) was assessed by observation by the individuals who conducted the interviews, with possible categories “Male”, “Female”, “Don’t know”, “Missing: Other” and “No answer.”

***Size of town*** (size_5c) was estimated among the following categories: “under 2000”, “2–5,000”, “5–10,000”, “10–20,000”, “20–50,000”, “50–100,000”, “100–500,000”, “500,000 and more.”

***Highest educational level attained*** (X025A_01) was chosen among the following categories: “Less than primary”, “Primary”, “Lower secondary”, “Upper secondary”, “Post-secondary non tertiary”, “Short-cycle tertiary”, “Bachelor or equivalent”, “Master or equivalent”, “Doctoral or equivalent.”

***Income*** (X047_WVS7) was assessed on a 10-point response scale range, with higher values representing higher income group, described as “steps.”

***Employment status*** (X028) was assessed with the question “Are you yourself gainfully employed at the moment or not? Please select from the card the employment status that applies to you.” and possible answers “Full time (30h a week or more)”, “Part time (less then 30 hours a week)”, “Self employed”, “Retired/pensioned”, “Housewife (not otherwise employed)”, “Student”, “Unemployed”, “Other.”

### Country-level variables

Individualism–collectivism and flexibility–-monumentalism scores were sourced from Minkov and Kaasa [26], with all inhabitants of a given country assigned the corresponding country-level scores on these dimensions.

### Data analysis plan

First, we dummy-coded singlehood variable (‘X007 Marital status’), so that 0 represented individuals at the time of the study in romantic and committed relationships (i.e., ‘Married’ and ‘Living together as married’), and 1–single individuals (i.e., ‘Single/Never married’). Those who were, at the time of the study, divorced, separated, and widowed, were excluded from the main analyses. We also dummy-coded unemployment variable (‘X028 - Employment status Respondent’), so that 0 represented those not officially unemployed (i.e., ‘Full time (30h a week or more)’, ‘Part time (less than 30 hours a week)’, ‘Self employed’, ‘Retired/pensioned’, ‘Housewife (not otherwise employed)’, ‘Student’, ‘Other’) and 1–those officially unemployed (‘Unemployed’).

Second, we group-mean centered participants’ age (‘X003 – Age’), size of town (‘size_5c’), highest attained educational level (‘X025A_01 - Highest educational level attained - Respondent: ISCED‐code one digit’), and income (‘X047_WVS7 - Scale of incomes (WVS7)’), and grand-mean centered cultural variables (i.e., Minkov-Hofstede

individualism-collectivism and flexibility-monumentalism).

Third, we determined the percentage of singe adults across countries included in the analyses.

Fourth, we performed generalized linear mixed-effects analyses with the logit link function. We nested participants within countries. The binary singlehood variable was regressed on participants’ sex, age, size of town, education, income, employment status and country-level individualism-collectivism and flexibility-monumentalism. In the second model, we tested for cross-level interactions between individual- and country-level variables. In the third model, we freed individual-level slopes. We then compared the models with the Bayesian Information Criterion (BIC), Akaike Information Criterion (AIC), and likelihood-ratio tests (LRT). The recommended guidelines were adhered to, that is, the change in the BIC between the two models (when ΔBIC > 10, the latter model indicates a better fit, the change in the AIC between the two models (similarly as in the BIC), and the significance of the LRT. Finally, to measure the amount of potential multicollinearity in our models, we analyzed Variance Inflation Factor (VIF) [31]. Following recommended guidelines, VIF values > 5 were regarded as indicating potential multicollinearity that may warrant concern [32,33].

The generalized linear mixed-effects modelling approach we used allowed us to model the binary outcome (singlehood), account for the non-independence of observations and between-country heterogeneity by nesting participants within countries, and estimate both fixed and random effects for the predictors [34]. This framework is particularly well suited to testing cross-level moderation because it permits country-level variation in intercepts and slopes while producing correct standard errors for clustered data [35]. All the analyses were performed in R (Version 4.4.1). The analysis code has been provided in the information in S1 Text in the S1 File.

## Results

In the preliminary step of analyses, we determined the percentage of single adults across 59 countries included in the analyses. In the second step, we assessed the models fit to the data (see Table 1).

**Table 1:** Comparison of the models’ fit between Model 1 including all predictors, Model 2 including all predictors and interactions, and Model 3 including all predictors, interactions, and freed slopes.

	Model 1	Model 2	Model 3
BIC	57091.91	55474.38	54148.88
AIC	57000.82	55273.97	53702.51
LRT (*χ*^*2*^)		1750.8***	1625.5***

*Note*. Smaller values of BIC and AIC suggest that a given model fits data better.

*** *p* < .001.

As Table 1 displays, according to the BIC, AIC, and LRT comparisons, each subsequent model showed better fit to the data. Therefore, in the manuscript we show the results of the most advanced model that had the best fit, that is, the model with the cross-level interactions and freed individual-level slopes (see Table 2). Importantly, we did not observe evidence for potential issues with multicollinearity, as all VIFs were below 5 (Max = 3.46, *M* = 2.32, *SD* = 0.57).

**Table 2:** Results of the generalized linear mixed-effects model with the dichotomized variable representing being a single (0 – non-single, 1 – single) on variables of interest with participants nested within countries.

Fixed effects	*Log-Odds*	SE	95% CI	*p*
**Individual-level predictors**				
Age	‒1.788	0.086	[-1.957, -1.619]	<.001
Sex^a^	‒0.261	0.029	[-0.318, -0.203]	<.001
Size of town	0.115	0.020	[0.075, 0.155]	<.001
Education	0.160	0.029	[0.103, 0.216]	<.001
Income	‒0.12	0.029	[-0.178, -0.062]	<.001
Unemployment^b^	0.221	0.020	[0.182, 0.259]	<.001
**Country-level predictors**				
Individualism^c^	0.418	0.135	[0.154, 0.683]	.002
Flexibility^d^	‒0.460	0.132	[-0.720, -0.201]	<.001
**Cross-level interactions**				
Age × Individualism	0.576	0.105	[0.371, 0.782]	<.001
Age × Flexibility	‒0.307	0.103	[-0.508, -0.106]	.003
Sex × Individualism	0.131	0.036	[0.061, 0.200]	<.001
Sex × Flexibility	‒0.01	0.035	[-0.079, 0.058]	.769
Size of town × Individualism	0.012	0.024	[-0.034, 0.059]	.601
Size of town × Flexibility	‒0.006	0.023	[-0.050, 0.039]	.800
Education × Individualism	‒0.094	0.035	[-0.163, -0.025]	.008
Education × Flexibility	‒0.013	0.034	[-0.081, 0.054]	.697
Income × Individualism	‒0.190	0.036	[-0.261, -0.119]	<.001
Income × Flexibility	‒0.038	0.034	[-0.104, 0.028]	.262
Unemployed × Individualism	‒0.071	0.024	[-0.118, -0.025]	.003
Unemployed × Flexibility	0.072	0.026	[0.022, 0.122]	.005
**Random Effects**	**Variance**	***SD***		
Intercept	0.946	0.973		
Age	0.421	0.649		
Sex^a^	0.159	0.399		
Size of town	0.013	0.113		
Education	0.012	0.109		
Income	0.011	0.103		
Unemployment^b^	0.168	0.410		

*Note*. ^a^ Men as a reference group. ^b^ Employed individuals as a reference group, ^c^ Minkov-Hofstede dimensions Individualism-collectivism, with higher values representing higher individualism, ^d^ Minkov-Hofstede dimensions Flexibility-monumentalism, with higher values representing higher flexibility, *ICC* = 0.223, Pseudo marginal *r*^2^ = 0.487, df_residuals_ = 66754, deviance = 53604.5, all VIFs below 3.46 (**M* *= 2.32, *SD* = 0.57).

All the individual-level socio-demographic characteristics were significantly related to being single. To be precise, higher likelihood of being single was related to being younger, being male, living in a larger town, having a higher educational level and a lower income, and being unemployed. Individual-level variables explained 38.6% of the variance in single status. Further, country-level individualism-collectivism and flexibility-monumentalism dimensions also emerged to be significant predictors of being single. Specifically, higher likelihood of being single was related to the origin from countries with higher scoring on individualism and lower scoring on flexibility dimension. However, these country-level variables explained only 5.13% of the variance in single status.

**Fig 1:**
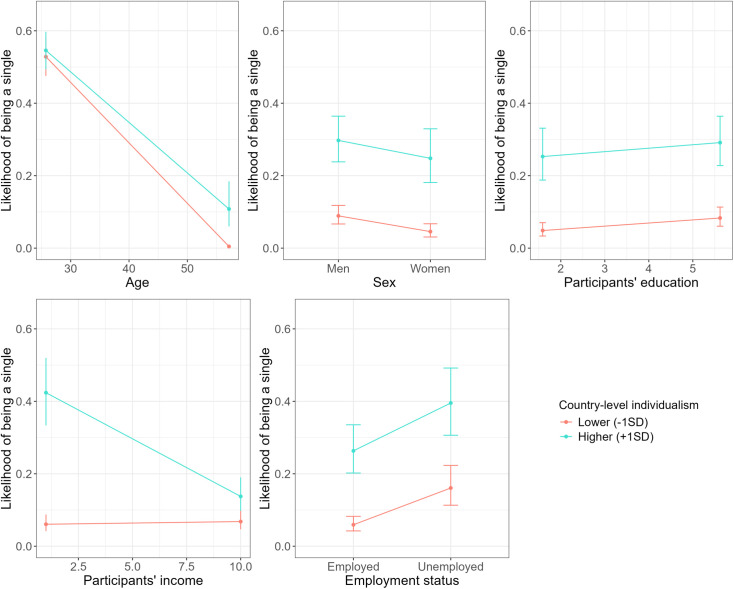
The likelihood of being single depending on country-level individualism and individual-level demographic variables (i.e., age, sex, education, income, and employment status).

**Fig 2:**
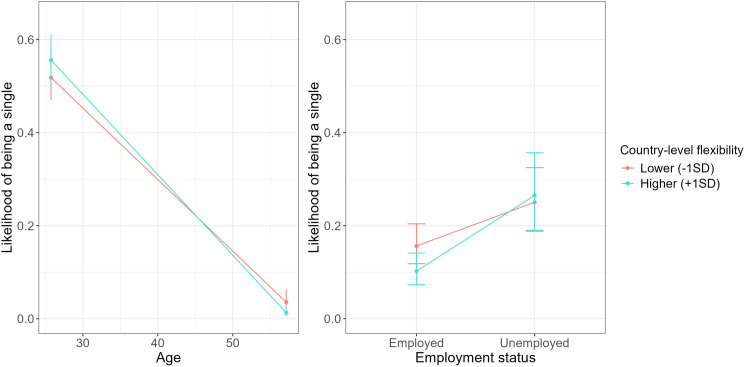
The likelihood of being single depending on country-level flexibility and individual-level demographic variables (i.e., age and employment status).

Finally, the performed analysis revealed several significant cross-level interactions between the individual-level socio-demographic characteristics and country-level individualism-collectivism and flexibility-monumentalism dimensions (see Table 2 and Figs 1 and 2).

As Table 2 and Figs 1 and 2 display, the likelihood of being single differed regarding country-level individualism and participants’ age, sex, education, income, and employment status, and between country-level flexibility and participants’ age and employment status.

To be precise, the increase in the odds of being single was more pronounced with younger (vs. older) age in less individualistic countries compared to more individualistic countries. Among all interactions, those involving age were the strongest. Moreover, the effect size for individualism was larger than that for flexibility. A higher likelihood of being single was more pronounced for older (vs. younger) individuals in less flexible countries. The sex difference in the odds of being single was more evident in more (vs. less) individualistic countries. In more individualistic countries being male was related to higher likelihood of being single compared to being female. This was the second largest effect among all interactions. There were larger differences in the odds ratio of being single as a function of education in less (vs. more) individualistic countries. Higher likelihood of being single was related to higher education levels in more individualistic countries compared to less individualistic countries. This was the third largest effect among all interactions.

Further, we observed the differences in the odds ratio of being single depending on participants’ income in more individualistic countries. To be precise, higher likelihood of being single was related to lower income in more individualistic countries, but such an association was not observed in more collectivistic countries. Finally, the increase in the odds of being single was more pronounced for unemployed (vs. employed) individuals from less (vs. more) individualistic and more (vs. less) flexible countries. Importantly, except for the interactions between age and the cultural variables individualism and flexibility, which were relatively large, the other significant interactions involving cultural variables were small or negligible. All fixed effects (i.e., individual- and country-level variables and their cross-level interactions) explained 48.7% of the variance in single status, while the full model including random effects explained 61.54%. The intraclass correlation coefficient (ICC) indicated that 22.3% of the variance in single status was attributable to between-country differences.

In the final step, we re-ran the model with interactions and random slopes, using an alternative operationalization of singlehood. Specifically, divorced, widowed, and separated individuals were included in the analysis and classified as single. This analysis produced the same pattern of results as those reported above (see Table S2 in the S1 File).

## Discussion

In our study, we formulated one broad open research question: How do sociodemographic characteristics (age, sex, size of town, education, income, employment) measured at the individual level relate to the rates of single adults across countries depending on country-level spectra of individualism–collectivism and flexibility–monumentalism? In line with past research (e.g., 2,3), the results of our study provided evidence that, indeed sociodemographic and cultural factors are important in predicting the prevalence of single adults across many countries, and that their links with singlehood may depend on cultural-level factors.

First, the performed analyses showed that all sociodemographic factors measured at the individual level were significantly related to the singlehood status across countries. We found that being younger was related to a greater likelihood of being single. This pattern is fully understandable given that individuals at younger ages and in earlier phases of human development are in a period of searching for and selecting a lifetime partner. For younger people, remaining single may be more normative than in later life. In addition, past studies have indicated that with increasing age, chances of transitioning from a single status to other types of relationship statuses (e.g., married or cohabitating) increase [15,36]. As a result, since the chances of forming a relationship increase with age, the chances of remaining single decrease.

Regarding sex, we determined that being male was related to a greater likelihood of being single. This finding might be explained by the fact that, in general, women are considered to be more relational than men are and to value social connectedness more than men are [16]. In turn, men are generally considered to be more oriented toward activities than toward being dependent in relationships [16]. Evolutionarily, women might be more eager to set the relationship in stone to secure the resources a potential long-term committed partner may provide [37]. Thus, women may report not being single compared to men reporting being single, even though they would hypothetically be in a similar position.

In addition, women are believed to experience greater social pressure to find a partner and cohabitate or marry [17]. Therefore, women may be less likely to be single than men are, as they are considered to experience greater internal motivation and/or external social pressure to find a partner and enter into a relationship. At the same time, it is probable that the observed in the current analyses gender difference in reported single status between men and women is an illusory difference created by a greater likelihood of reporting by women than men that they are in marriage-like relationship analogically as in the case of gender differences in reported sex partners between men and women arising from the differences in attitudes related to sexual success (i.e., the biases in reported sexual behaviors) [38].

With respect to the size of the town, contrary to the notion in the literature that more compact urban places may offer people more opportunities to meet new people than low-density suburban neighborhoods [13], we observed that living in a larger town was related to a greater likelihood of being single. This finding may be explained by another notion in the literature that urban residence is related to lower adherence to traditional values [39]. Since single status may be considered a less traditional life pathway [4,7,12], urban residences with greater opportunities and facilities may prompt living alone.

Regarding education, we observed that having a higher education level was associated with a greater likelihood of being single. These findings corroborate past studies showing that completing education requires time and delays entry into the labor market, leading to the postponement of union formation [3,8].

In connection with the above findings, lower income and unemployment were found to be related to a greater likelihood of being single. These findings corroborate past studies showing that a lack of economic well-being and stability may deter people from entering into serious relationships [3,9,10]. Notably, earnings were found among men to be positively linked with entry into cohabitation or marriage, and men with better long-term socioeconomic prospects had greater chances of marrying [40].

Second, the results also provided evidence for a greater likelihood of being single in countries characterized by a higher level of individualism and a lower level of flexibility. These findings supports the findings of past literature, suggesting that stronger adherence to individualistic values, lower dependence on the family, and escalating attempts at self-realization may contribute to a greater incidence of singlehood [3,7]. In addition, the essential traits of individualism, which involve, among others, freedom to behave in line with one’s own beliefs and to decide which societal rules to abide by, particularly in the domains of religion, sexuality, the creation and termination of life, and traditional gender roles, may be considered enhancing freedom in choosing and living a single life in line with an individual’s desires and decisions and increasing rates of voluntary singlehood [4,12].

Furthermore, the link between a higher level of individualism and lower flexibility seems surprising since flexible cultures enhance the importance of adaptability and modesty and self-reliance and independence [22]. These traits might have appeared to prompt the increase in the number of single people living alone. On the other hand, flexibility (corresponding to Hofstede’s long-term orientation) highlights self-control and suppression of desire for immediate gratification and prompts slow LHS (life-history strategies) [27]; both FLX and a slow LHS are considered to be prerequisites for long-term goals, such as obtaining good education [27]. As a result, less-flexible countries shifting toward greater monumentalism can be considered countries adhering to the desire to feel good about oneself [27] and prompting rather rapid rather than slow LHS. In turn, individuals with faster LHS are supposed to have numerous sexual partners, a lower investment in parenting and engage in short, not long-term, relationships [41]. These traits of less-flexible countries might enhance the tendency to live as a single person, contributing to higher shares of single adults in these countries than in more-flexible countries characterized by slower LHS, which is, in turn, recognized to be related to stronger investment and commitment in romantic relationships and parenting [41].

Importantly, the performed analyses demonstrated the existence of several interactions between the selected sociodemographic characteristics and country-level measures of Minkov-Hofstede individualism–collectivism and flexibility–monumentalism dimensions. These results imply that the salience of certain individual sociodemographic characteristics (age, sex, size of town, education, income, employment) may differ across countries as a function of the features specific for individualism–collectivism and flexibility–monumentalism.

Regarding age, sex, education, and employment as a function of the country-level individualism–collectivism dimension, we unveiled a coherent constellation of mutually connected factors related to the rates of single adults. Specifically, in more-individualistic countries, we found that a higher likelihood of being single was related to younger age, male identity, a higher education level, unemployment and lower income.

Interpreting the results holistically in regard to Minkov’s individualism–collectivism dimension, it might be inferred that 1) more-individualistic countries may expect their young members to acquire resources before entering serious relationships, allowing them to develop an independent and self-reliant style of life. These resources may involve completing education (what currently happens at a later age due to its elongation) [3] and obtaining socioeconomic resources in terms of earning and employment [9]. In turn, possessing these resources may be critical for young people to leave their family of origin and run a one-person household [3,9]. Thus, when people have more financial resources to live alone (SALA; single and living alone; [36], the prevalence of young single people may increase.

The observed link between male identity and a greater likelihood of remaining single in more-individualistic countries may suggest the importance of independent and self-reliant lifestyles in these countries. In turn, this emphasis on independence, self-reliance and an independent construal of self by becoming and staying independent from others [42] may align with independence and self-reliance, which are personality traits ascribed to single men in traditional social perception. In turn, less-individualistic countries may reinforce more interdependent construals of the self in which connectedness is fundamental and the self is meaningful and complete due to embeddedness in social relationships [42].

Regarding Minkov-Hofstede flexibility–monumentalism, we determined more pronounced links between a higher likelihood of being single, older, and unemployed in less-flexible countries than in more-flexible countries. To recall, lower country flexibility means greater monumentalism, which has core traits that involve an invariant and proud self, the importance of feeling good and following one’s own desires and impulses [26]. In addition, more-monumentalist countries emphasize the role of exchanging services and help [26]. In turn, older individuals are believed to be characterized by lower conformity to societal influences and to lead a more authentic life based on their intrinsic values and beliefs [43]. In light of these traits, it is plausible to assume that a greater number of individuals in less-flexible (more monumentalist) countries may live single as they age since such countries reinforce living in line with one’s desires (e.g., living as a single person) instead of with social expectations (such as being in a serious relationship). These circumstances are considered important for older individuals. Moreover, due to the emphasis in monumentalist countries on providing help and services to others, it is also probable that older people may choose to live solo as in these societies, they can rely on help and support from other people.

The more pronounced association between singlehood and a higher likelihood of being unemployed in less-flexible countries appears to be puzzling. On the one hand, since more-flexible countries enhance independence and greater independence has been observed in societies with higher rates of highly educated individuals who are able to obtain good jobs and take care of themselves [27], it might be assumed that a greater number of single people would be observed in more- rather than less-flexible countries. This is because having a good job and high income may be critical for enabling young people to leave their family of origin and run a one-person household [3,9]. Thus, when people have more financial resources to live alone [36], the prevalence of single people may increase.

On the other hand, well-educated individuals with good jobs in more-flexible countries probably possess more financial resources, allowing them to enter into relationships and start families, while a lack of economic well-being and stability discourages people from establishing serious relationships [3,9,10]. In contrast, if less-flexible countries deemphasize the importance of independence, higher education, higher income, and possession of a good job, individuals may encounter financial and economic obstacles (due to being unemployed) to finding a partner and establishing a serious relationship, which contributes to increased rates of singlehood. Indeed, among men in particular, men’s employment is considered to be important for families, as it makes them breadwinners [44].

This study has several strengths, especially the large sample size and countries’ cultural, history, and language differences, which contributed to the samples’ diversity. Our analyses significantly contributed to the literature by showing the interactions between the sociodemographic factors measured at the individual level and cultural factors measured at the country level in terms of Minkov’s individualism–collectivism and flexibility–monumentalism, which represent a revised variant of Hofstede’s subjective-culture model in regard to singlehood rates. Specifically, we demonstrated that Minkov-Hofstede individualism–collectivism and flexibility–monumentalism which have been shown in past literature to be linked to various social practices such as transparency-corruption, political and economic freedom, competitiveness, innovation output, fatalities in transport and industry, gender equality, economic equality, educational achievement, working hours, and violent crime [26] might also be related to the prevalence of adult singlehood. Our findings highlight the necessity to consider both the sociodemographic and cultural factors in regard to singlehood rates, as the joint analyses of these factors may offer a deeper and more comprehensive understanding of singlehood prevalence across countries. In other words, the analysis of singlehood phenomena only at the individual level may not be complete without considering the broader social and cultural contexts in which single individuals function.

### Limitations and future research directions

The current study is not without limitations. First, the correlational nature of the data derived from the World Values Survey [30] precludes the determination of the direction of the cause-and-effect links between sociodemographic characteristics and country-level individualism–collectivism and flexibility–monumentalism dimensions and rates of single adults. We may assume that, for instance, lower income, unemployment, higher country-level individualism and lower flexibility increase the likelihood of being single. However, reverse causality is not implausible. Living as a single person may lead to a decrease in an individual’s income, and individuals may become unemployed. Additionally, we cannot rule out the hypothesis that, because of living as a single, independent and self-reliant person and not uncommonly choosing a single status in line with their personal desires, some people in individualistic and less-flexible countries may overestimate their independence and self-reliance and lead their lives according to their personal choices and decisions. Finally, individual sociodemographic and country-level characteristics may be associated with complex two-way causes and effects, reinforcing each other.

Second, our analyses focused merely on single/never-married individuals. Since single individuals are a very heterogeneous group differing, among other variables, in regard to relational history [2,4,5], future research may incorporate more diverse groups of single individuals with varying relational trajectories (e.g., individuals who became single as a result of the dissolution of a relationship). In addition, since the utilized samples were cross-sectional, we were not able to track further relationship trajectories of single individuals or whether they remained single or transited into an informal or marital relationship. To explore the dynamics of single adults’ relationship trajectories, it will be important for future research to expand the present cross-sectional findings through a longitudinal design.

Third, in the present analyses, we focused on the selected sociodemographic and country-level characteristics as related to the proportions of single adults. Although the current investigation focused on two well-recognized and solid cultural dimensions, they represent only two of many cultural dimensions identified and discussed in the literature [26]. Therefore, another important task for future research would be to explore the role of other cultural dimensions in the prevalence of singlehood, such as relational mobility or Inglehart-Welzel model [45], which constitutes a socioecological factor defining the extent to which societies or groups afford their members freedom and opportunity to choose interpersonal relationships based on their preferences. Moreover, the present analyses omitted the issue of psychosocial functioning in single individuals. Therefore, it would be crucial to include psychological phenomena such as reasons for singlehood and life outcomes in future studies alongside sociodemographic and cultural characteristics.

## Conclusion

In conclusion, our analyses across 59 countries focused on how individual sociodemographic characteristics and country-level traits, measured in terms of individualism–collectivism and flexibility–monumentalism, operate in tandem and relate to the prevalence of single adults. Our findings suggest that demographic factors are only one side of the phenomenon of singlehood and that the cultural side is equally important and appears to operate in tandem with sociodemographic factors. This important analysis lays the groundwork for further exploration of the joint operation of individual and cultural characteristics behind the increasing prevalence of singlehood in many countries.

## Supporting information

S1 File Supplementary information contains Tables S1 and Table S2, which include characteristics of the sample and the results of the generalized linear mixed-effects model using a dichotomized variable representing single status, along with the analysis code. Marta Kowal, Ph.D. https://orcid.org/0000-0001-9050-1471 Katarzyna Adamczyk, Ph.D. https://orcid.org/0000-0002-7612-8380
(DOCX)
