# Supplementary material for: Sociodemographic and cultural factors are related to singlehood rates: A multilevel analysis across 59 countries from the World Values Survey
Source: PLoS One. 2025 Oct 29;20(10):e0335416. doi: 10.1371/journal.pone.0335416 (PMC12571265; doi:10.1371/journal.pone.0335416)
Supplement: S1 File — Supplementary information contains Tables S1 and Table S2, which include characteristics of the sample and the results of the generalized linear mixed-effects model using a dichotomized variable representing single status, along with the analysis code. Marta Kowal, Ph.D. https://orcid.org/0000-0001-9050-1471 Katarzyna Adamczyk, Ph.D. https://orcid.org/0000-0002-7612-8380 (DOCX) [file pone.0335416.s001.docx]

**Supplementary Material**

| **Table S1**  *Characteristics of the sample* | | | | | | | | |
| --- | --- | --- | --- | --- | --- | --- | --- | --- |
| **Country** | ***n*** | **Number of singles (%)** | **Number of men (%)** | **Mean age (*SD*)** | **Mean size of town (*SD*)** | **Mean education (*SD*)** | **Mean income (*SD*)** | **Number of unemployed (%)** |
| Andorra | 716 | 195 (16.1%) | 378 (52.8%) | 44.46 (15.18) | 2.09 (0.65) | 3.83 (1.96) | 5.53 (1.68) | 9 (1.3%) |
| Argentina | 715 | 254 (22.3%) | 356 (49.8%) | 38.99 (15.98) | 4.07 (1.41) | 2.83 (1.44) | 5.05 (1.61) | 21 (2.9%) |
| Armenia | 930 | 158 (28.3%) | 340 (36.6%) | 45.41 (15.96) | 2.94 (1.67) | 4.42 (1.7) | 4.77 (1.99) | 117 (12.6%) |
| Australia | 1325 | 277 (20.3%) | 559 (42.2%) | 51.28 (16.52) | 3.64 (1.42) | 4.83 (1.69) | 5.46 (1.99) | 44 (3.3%) |
| Bangladesh | 1177 | 143 (17.2%) | 592 (50.3%) | 36.35 (12.79) | 1.77 (0.75) | 1.86 (1.64) | 5.6 (2.07) | 46 (3.9%) |
| Brazil | 1306 | 470 (26.4%) | 637 (48.8%) | 40.63 (16.05) | 3.92 (1.13) | 2.88 (1.65) | 4.08 (2.14) | 208 (15.9%) |
| Canada | 3531 | 1093 (22.6%) | 1854 (52.5%) | 45.02 (16.6) | 3.42 (1.4) | 4.92 (1.6) | 5.67 (1.81) | 215 (6.1%) |
| Chile | 711 | 114 (12%) | 360 (50.6%) | 44.65 (14.64) | 3.78 (1.31) | 3.98 (1.59) | 4.77 (1.7) | 24 (3.4%) |
| China | 2669 | 369 (16.2%) | 1227 (46%) | 43.81 (14.15) | 3.33 (0.51) | 2.93 (1.91) | 4.19 (1.84) | 117 (4.4%) |
| Colombia | 1295 | 510 (30.4%) | 677 (52.3%) | 36.88 (14.84) | 3.63 (1.22) | 3.18 (1.77) | 4.46 (2.52) | 141 (10.9%) |
| Cyprus | 798 | 206 (27%) | 412 (51.6%) | 43.12 (15.75) | 2.33 (1.25) | 4.53 (2.29) | 5.36 (1.65) | 49 (6.1%) |
| Czechia | 892 | 228 (29.9%) | 440 (49.3%) | 44.91 (15.73) | 2.42 (1.4) | 3.25 (1.77) | 5.45 (1.61) | 18 (2%) |
| Ecuador | 1021 | 360 (17.1%) | 500 (49%) | 37.99 (15.43) | 3.36 (1.42) | 3.23 (1.74) | 4.83 (2.19) | 213 (20.9%) |
| Egypt | 975 | 206 (27.4%) | 551 (56.5%) | 38.03 (12.98) | 3.21 (1.22) | 2.79 (2.03) | 5.08 (1.37) | 46 (4.7%) |
| Ethiopia | 1132 | 319 (12.7%) | 606 (53.5%) | 31.3 (11.36) | 2.18 (0.74) | 2.09 (1.87) | 4.42 (2.23) | 93 (8.2%) |
| Germany | 1234 | 270 (13.3%) | 636 (51.5%) | 48.51 (17.28) | 2.81 (1.25) | 4.14 (1.77) | 5.28 (1.67) | 56 (4.5%) |
| Greece | 901 | 238 (34.4%) | 461 (51.2%) | 47.85 (17.08) | 3.18 (1.64) | 3.4 (1.84) | 4.66 (1.71) | 121 (13.4%) |
| Guatemala | 994 | 547 (26.5%) | 495 (49.8%) | 32.11 (13.19) | 4.18 (1) | 4.17 (2.28) | 6 (2.01) | 69 (6.9%) |
| Hong Kong | 1849 | 638 (25.6%) | 895 (48.4%) | 45.62 (15.22) | 5 (0) | 4.2 (1.85) | 4.9 (1.73) | 46 (2.5%) |
| Indonesia | 2862 | 409 (20.6%) | 1368 (47.8%) | 38.83 (12.89) | 1.75 (0.88) | 2.31 (1.53) | 4.28 (2.4) | 94 (3.3%) |
| Iraq | 1110 | 258 (26.3%) | 574 (51.7%) | 36.14 (13.14) | 3.93 (1.39) | 2.92 (2.02) | 4.47 (1.85) | 108 (9.7%) |
| Ireland | 274 | 76 (30.4%) | 137 (50%) | 48.81 (16.7) | 3.22 (0.68) | 4.15 (2.01) | 5.64 (2.02) | 10 (3.6%) |
| Japan | 1032 | 123 (18.4%) | 501 (48.5%) | 53.73 (16.79) | 4.1 (0.88) | 4.47 (1.47) | 4.47 (2.65) | 5 (0.5%) |
| Jordan | 1072 | 227 (25.8%) | 592 (55.2%) | 42.09 (14.6) | 3.42 (1.58) | 3.28 (1.72) | 4.1 (2.03) | 73 (6.8%) |
| Kazakhstan | 922 | 173 (26%) | 445 (48.3%) | 39.59 (12.98) | 3.35 (1.47) | 4.93 (1.68) | 5.61 (1.64) | 45 (4.9%) |
| Kenya | 919 | 425 (27%) | 479 (52.1%) | 30.3 (9.7) | 2.69 (1.37) | 3.33 (1.67) | 4.61 (1.98) | 222 (24.2%) |
| Korea (the Republic of) | 1210 | 311 (31.3%) | 596 (49.3%) | 45.1 (14.81) | 4.64 (0.62) | 4.17 (1.58) | 4.89 (1.35) | 18 (1.5%) |
| Kyrgyzstan | 983 | 192 (15%) | 414 (42.1%) | 38.96 (13.98) | 2.4 (1.45) | 4.45 (1.72) | 5.13 (2.16) | 37 (3.2%) |
| Lebanon | 1144 | 425 (22.9%) | 583 (51%) | 40.06 (15.02) | 2.89 (1.03) | 3.73 (1.94) | 5.55 (1.82) | 52 (4.9%) |
| Libya | 1064 | 355 (21.4%) | 563 (52.9%) | 39.87 (12.79) | 2.84 (1.33) | 4.35 (1.64) | 5.44 (1.92) | 12 (1.6%) |
| Macao | 758 | 314 (49.5%) | 360 (47.5%) | 39.73 (16.41) | 5 (0) | 4.01 (1.97) | 5.04 (1.42) | 45 (3.6%) |
| Malaysia | 1264 | 432 (31%) | 647 (51.2%) | 37.91 (13.1) | 2.42 (1.31) | 3.37 (1.71) | 4.62 (2.05) | 88 (6.4%) |
| Mexico | 1381 | 261 (26.4%) | 736 (53.3%) | 41.32 (15.53) | 2.85 (1.57) | 3.13 (1.62) | 4.31 (2.37) | 114 (7.6%) |
| Mongolia | 1505 | 341 (19.8%) | 753 (50%) | 37.68 (13.6) | 3.84 (1.61) | 4.7 (1.67) | 5.52 (1.84) | 122 (11%) |
| Morocco | 1106 | 440 (13%) | 572 (51.7%) | 36.7 (13.47) | 3.34 (1.39) | 2.53 (2.16) | 5.21 (1.66) | 66 (5.9%) |
| Myanmar | 1114 | 245 (29.6%) | 571 (51.3%) | 39.55 (14.04) | 1.53 (0.63) | 2.42 (1.57) | 4.71 (2.01) | 52 (3.7%) |
| Netherlands (the) | 1394 | 360 (22.3%) | 697 (50%) | 52.43 (15.52) | 3.46 (0.75) | 4.87 (1.83) | 6.12 (2.63) | 35 (4.6%) |
| New Zealand | 759 | 111 (24.9%) | 342 (45.1%) | 55.04 (15.71) | 3.03 (1.41) | 4.64 (1.61) | 6.12 (2.77) | 212 (19.7%) |
| Nicaragua | 1077 | 389 (28.6%) | 540 (50.1%) | 33.93 (13.42) | 2.96 (0.91) | 2.79 (1.87) | 4.63 (2.5) | 208 (18%) |
| Nigeria | 1158 | 472 (12.9%) | 608 (52.5%) | 32.1 (11.72) | 2.08 (1.05) | 2.6 (1.88) | 4.42 (2.02) | 93 (5.1%) |
| Pakistan | 1824 | 277 (19.1%) | 948 (52%) | 35.09 (11.01) | 2.47 (1.66) | 2.19 (1.98) | 4.44 (2.3) | 56 (4.5%) |
| Peru | 1253 | 365 (17.3%) | 653 (52.1%) | 38.71 (14.69) | 3.07 (1.29) | 3.37 (1.69) | 5 (1.9) | 200 (18.5%) |
| Philippines (the) | 1080 | 195 (43.8%) | 567 (52.5%) | 41.82 (15.21) | 1.73 (0.82) | 2.4 (1.74) | 4.43 (2.06) | 92 (10.8%) |
| Puerto Rico | 853 | 290 (25%) | 365 (42.8%) | 46.77 (17.64) | 3.05 (0.43) | 4.55 (1.8) | 5.13 (2.24) | 31 (3.5%) |
| Romania | 893 | 156 (16.8%) | 372 (41.7%) | 45.75 (17.14) | 2.5 (1.35) | 3.29 (1.55) | 5.44 (1.92) | 51 (4.4%) |
| Russian Federation (the) | 1151 | 335 (24%) | 584 (50.7%) | 40.62 (15.28) | 3.31 (1.55) | 4.96 (1.74) | 5.04 (1.9) | 135 (17.3%) |
| Serbia | 782 | 229 (35.4%) | 375 (48%) | 43.57 (15.57) | 2.92 (1.3) | 5.39 (2.04) | 4.91 (1.92) | 106 (6%) |
| Singapore | 1759 | 574 (32.2%) | 840 (47.8%) | 46.05 (15.52) | 5 (0) | 4.41 (1.87) | 5.09 (1.63) | 37 (4.3%) |
| Slovakia | 862 | 203 (19.4%) | 432 (50.1%) | 49.33 (15.38) | 2.04 (1.09) | 3.43 (1.81) | 5.32 (1.78) | 39 (3.8%) |
| Taiwan (Province of China) | 1029 | 350 (39.5%) | 522 (50.7%) | 46.02 (16.47) | 3.49 (0.59) | 4.39 (1.94) | 4.65 (1.69) | 279 (25.4%) |
| Tajikistan | 1097 | 197 (32.9%) | 572 (52.1%) | 40.08 (14.89) | 1.84 (1.28) | 4.28 (1.83) | 5.69 (1.6) | 69 (5.1%) |
| Thailand | 1353 | 187 (22.1%) | 651 (48.1%) | 45.63 (13.04) | 1.6 (1.17) | 2.18 (1.68) | 4.77 (1.76) | 102 (9.9%) |
| Tunisia | 1027 | 319 (17.1%) | 512 (49.9%) | 41.52 (14.55) | 2.6 (1.21) | 2.71 (1.77) | 4.77 (2.02) | 149 (6.8%) |
| Turkey | 2191 | 748 (20.8%) | 1128 (51.5%) | 38.24 (12.5) | 3.72 (0.96) | 2.36 (1.91) | 5.37 (1.72) | 60 (6.2%) |
| Ukraine | 961 | 146 (28.9%) | 425 (44.2%) | 44.42 (15.28) | 2.71 (1.66) | 5.07 (1.6) | 4.78 (1.82) | 59 (3.7%) |
| United Kingdom of Great Britain and Northern Ireland (the) | 1584 | 434 (36.7%) | 735 (46.4%) | 48.32 (16.82) | 3.37 (1.04) | 4.48 (2) | 6.03 (2.14) | 119 (8.1%) |
| United States of America (the) | 1473 | 498 (20.4%) | 822 (55.8%) | 40.31 (15.43) | 3.74 (0.78) | 4.98 (1.59) | 5.1 (1.86) | 53 (7.7%) |
| Uruguay | 692 | 227 (25%) | 241 (34.8%) | 44.21 (16.59) | 3.59 (1.45) | 3.55 (2.02) | 5.26 (2.15) | 89 (8.7%) |
| Venezuela (Bolivarian Republic of) | 1026 | 396 (13.3%) | 510 (49.7%) | 36.45 (14.54) | 3.72 (1.15) | 3.15 (1.63) | 4.51 (1.85) | 0 (0%) |
| **Total** | 71169 | 19060 (26.8%) | 35308 (49.6%) | 41.74 (15.63) | 3.13 (1.45) | 3.62 (2.03) | 4.99 (2.05) | 5090 (7.15%) |

**Table S2**

Results of the generalized linear mixed-effects model with the dichotomized variable representing being a single (0 – non-single, including married or living together as married, 1 – single/never married, divorced, separated, widowed) on variables of interest with participants nested within countries.

| **Fixed effects** | **Log-Odds** | **SE** | **95% CI** | **p** |
| --- | --- | --- | --- | --- |
| **Individual-level predictors** |  |  |  |  |
| Age | -1.905 | 0.086 | [-2.074, -1.737] | < .001 |
| Sex ^a^ | -0.284 | 0.027 | [-0.338, -0.231] | < .001 |
| Size of town | 0.107 | 0.019 | [ 0.070, 0.145] | < .001 |
| Education | 0.166 | 0.028 | [ 0.111, 0.221] | < .001 |
| Income | -0.097 | 0.026 | [-0.148, -0.046] | < .001 |
| Unemployment ^b^ | 0.193 | 0.018 | [ 0.158, 0.229] | < .001 |
| **Country-level predictors** |  |  |  |  |
| Individualism ^c^ | 0.421 | 0.139 | [ 0.149, 0.694] | 0.002 |
| Flexibility ^d^ | -0.466 | 0.135 | [-0.731, -0.202] | < .001 |
| Cross-level interactions |  |  |  |  |
| Age × Individualism | 0.544 | 0.104 | [ 0.340, 0.748] | < .001 |
| Age × Flexibility | -0.291 | 0.102 | [-0.490, -0.092] | 0.004 |
| Sex × Individualism | 0.119 | 0.033 | [ 0.055, 0.184] | < .001 |
| Sex × Flexibility | -0.008 | 0.032 | [-0.071, 0.056] | 0.811 |
| Size of town × Individualism | 0.011 | 0.022 | [-0.032, 0.055] | 0.607 |
| Size of town × Flexibility | -0.004 | 0.021 | [-0.045, 0.038] | 0.865 |
| Education × Individualism | -0.099 | 0.034 | [-0.166, -0.032] | 0.004 |
| Education × Flexibility | -0.01 | 0.033 | [-0.075, 0.055] | 0.768 |
| Income × Individualism | -0.154 | 0.032 | [-0.216, -0.092] | < .001 |
| Income × Flexibility | -0.036 | 0.03 | [-0.094, 0.023] | 0.233 |
| Unemployed × Individualism | -0.064 | 0.022 | [-0.108, -0.021] | 0.003 |
| Unemployed × Flexibility | 0.058 | 0.023 | [ 0.013, 0.103] | 0.012 |
| **Random Effects** | **Variance** | **SD** |  |  |
| Intercept | 0.890 | 0.943 |  |  |
| Age | 0.392 | 0.626 |  |  |
| Sex a | 0.134 | 0.366 |  |  |
| Size of town | 0.011 | 0.104 |  |  |
| Education | 0.011 | 0.105 |  |  |
| Income | 0.008 | 0.088 |  |  |
| Unemployment ^b^ | 0.141 | 0.376 |  |  |

Note. ^a^ Men as a reference group. ^b^ Employed individuals as a reference group, ^c^ Minkov-Hofstede dimensions Individualism-collectivism, with higher values representing higher individualism, ^d^ Minkov-Hofstede dimensions Flexibility-monumentalism, with higher values representing higher flexibility, ICC = 0.213, Pseudo marginal *r^2^* = 0.492 *df_residuals_* = 76531, deviance = 56452.4, all VIFs below 3.44 (*M* = 2.31, *SD* = 0.57).

**Analysis code**

############### Singlehood European Values Survey & World Values Survey

## Load packages

library('dplyr')

library('kableExtra')

library("ggplot2")

library("lme4")

library('merTools')

library("parameters")

library("directlabels")

library("ggplot2")

# Load datasets

d <- read.csv2("EVS_WVS_Joint_csv_v4_0_short_ceesvau.csv",header=T,sep=";", dec ='.')

minkov <- read.csv2("Minkov_individualism_monumentalism_ceesvau.csv",header=T,sep=";", dec ='.')

## Merge minkov with d

d <- left_join(d, minkov, by='Country_live')

## Exclude those with missing relationship status

d <- d %>%

dplyr::filter(!is.na(Single01))

## Drop cases with missing Age

d <- d %>%

mutate(Age_original = Age) %>%

naniar::replace_with_na(replace = list(Age = c(-5, -4, -3, -2, -1)))

summary(d$Age)

table(d$Age)

### Investigate sample

# n across countries

kable(digits = 2,

d %>%

group_by(Country_live) %>%

tally() %>%

mutate(freq = paste0(round(100 * n/sum(n), 1), "%")) %>%

arrange(desc(n))) %>%

kable_styling(full_width = F)

## Employment across countries

kable(digits = 2,

d %>%

group_by(Country_live, Employment_recoded) %>%

tally() %>%

mutate(freq = paste0(round(100 * n/sum(n), 1), "%")) %>%

arrange()) %>%

kable_styling(full_width = F)

# Age

kable(digits = 2,

d %>%

group_by(Country_live) %>%

summarise(

m = mean(Age, na.rm=TRUE),

sd = sd(Age, na.rm=TRUE),

min = min(Age),

max = max(Age))

) %>%

kable_styling(full_width = F)

# Sex

kable(digits = 2,

d %>%

filter(complete.cases(Sex01)) %>%

group_by(Country_live, Sex01) %>%

tally() %>%

mutate(freq = paste0(round(100 * n/sum(n), 1), "%")) %>%

arrange()) %>%

kable_styling(full_width = F)

######## Compute singlehood rates

d <- d %>%

dplyr::group_by(Country_live) %>%

mutate(Singlehood_country = mean(Single01, na.rm=TRUE)*100)

summary(d$Singlehood_country)

colnames(d)

kable(digits = 2,

d %>%

filter(complete.cases(Singlehood_country)) %>%

group_by(Country_live) %>%

summarise(

m = mean(Singlehood_country, na.rm=TRUE))) %>%

kable_styling(full_width = F)

#### Create Country_live_factor

d <- d %>%

dplyr::mutate(Country_live_factor = factor(Country_live))

#### Create Unemployed01

d <- d %>%

dplyr::mutate(Unemployed01 = case_when(

Employment_recoded == "Full time (Self employed0h a week or more)" ~ 0,

Employment_recoded == "Other" ~ 0,

Employment_recoded == "Self employed" ~ 0,

Employment_recoded == "Student" ~ 0,

Employment_recoded == "Part time (less then Self employed0 hours a week)" ~ 0,

Employment_recoded == "Retired/pensioned" ~ 0,

Employment_recoded == "Housewife (not otherwise employed)" ~ 0,

Employment_recoded == "Unemployed" ~ 1,

.default = 99

))

table(d$Unemployed01)

d <- d %>%

naniar::replace_with_na(replace = list(Unemployed01 = c(99)))

########### Group standardize

d <- d %>%

dplyr::group_by(Country_live) %>%

dplyr::mutate(group_z_Age = scale(Age),

group_z_Size_of_town_clean = scale(Size_of_town_clean),

group_z_Education_clean = scale(Education_clean),

group_z_Income_clean = scale(Income_clean),

group_c_Age = scale(Age, scale = FALSE),

group_c_Size_of_town_clean = scale(Size_of_town_clean, scale = FALSE),

group_c_Education_clean = scale(Education_clean, scale = FALSE),

group_c_Income_clean = scale(Income_clean, scale = FALSE)

) %>%

dplyr::ungroup()

# Grand z

d <- d %>%

dplyr::mutate(grand_z_Individualism2022_MINKOV = scale(Individualism2022_MINKOV),

grand_z_Flexibility_2022_MINKOV = scale(Flexibility_2022_MINKOV))

# Create a backup

backupd <- d

### Choose only WVS

d <- d %>%

dplyr::filter(study_recoded == "WVS")

#### Create nomiss dataset

nomiss <- d %>%

dplyr::filter(!is.na(Single01) & !is.na(Age) & !is.na(Sex01) &!is.na(Size_of_town_clean) &!is.na(Education_clean) & !is.na(Income_clean) &!is.na(Unemployed01) &!is.na(Individualism2022_MINKOV) &!is.na(Flexibility_2022_MINKOV))

#### For additional check – Revision 1 PLOS

additionalanalyses <- nomiss

### Choose only currently single or not-single

d <- d %>%

dplyr::filter(Relationship_status_recoded == "Living together as married" | Relationship_status_recoded == "Married" | Relationship_status_recoded == "Single/Never married")

########################################################################################################

########################################################################################################

################################# Multilevel models #########################################

########################################################################################################

########################################################################################################

############### Minkov

glmer_model_all_interactions_slope <- lme4::glmer(Single01 ~ 1 +

group_z_Age + Sex01 + group_z_Size_of_town_clean + group_z_Education_clean + group_z_Income_clean + Unemployed01 +

grand_z_Individualism2022_MINKOV + grand_z_Flexibility_2022_MINKOV +

group_z_Age * grand_z_Individualism2022_MINKOV +

group_z_Age * grand_z_Flexibility_2022_MINKOV +

Sex01 * grand_z_Individualism2022_MINKOV +

Sex01 * grand_z_Flexibility_2022_MINKOV +

group_z_Size_of_town_clean * grand_z_Individualism2022_MINKOV +

group_z_Size_of_town_clean * grand_z_Flexibility_2022_MINKOV +

group_z_Education_clean * grand_z_Individualism2022_MINKOV +

group_z_Education_clean * grand_z_Flexibility_2022_MINKOV +

group_z_Income_clean * grand_z_Individualism2022_MINKOV +

group_z_Income_clean * grand_z_Flexibility_2022_MINKOV +

Unemployed01 * grand_z_Individualism2022_MINKOV +

Unemployed01 * grand_z_Flexibility_2022_MINKOV +

(1 + group_z_Age + Sex01 + Size_of_town_clean + Education_clean + Income_clean + Unemployed01| Country_live_factor),

binomial(link = "logit"),

data = d)

summary(glmer_model_all_interactions_slope)

parameters::model_parameters(glmer_model_all_interactions_slope, standardize = "refit", digits = 3, ci_digits = 3)

jtools::summ(glmer_model_all_interactions_slope, digits = 3)

# VIFs

car::vif(glmer_model_all_interactions_slope)

emmeans::emmeans(glmer_model_all_interactions_slope, list(pairwise ~ Sex01 * grand_z_Individualism2022_MINKOV), pbkrtest.limit = 100000, adjust = "tukey")

# Odds ratio

exp(lme4::fixef(glmer_model_all_interactions_slope))

## First model

glmer_model_all <- lme4::glmer(Single01 ~ 1 + group_z_Age + Sex01 + group_z_Size_of_town_clean + group_z_Education_clean + group_z_Income_clean + Unemployed01 +

grand_z_Individualism2022_MINKOV + grand_z_Flexibility_2022_MINKOV +

(1 | Country_live_factor), binomial(link = "logit"),

data = d)

## Second model

glmer_model_all_interactions <- lme4::glmer(Single01 ~ 1 +

group_z_Age + Sex01 + group_z_Size_of_town_clean + group_z_Education_clean + group_z_Income_clean + Unemployed01 +

grand_z_Individualism2022_MINKOV + grand_z_Flexibility_2022_MINKOV +

group_z_Age * grand_z_Individualism2022_MINKOV +

group_z_Age * grand_z_Flexibility_2022_MINKOV +

Sex01 * grand_z_Individualism2022_MINKOV +

Sex01 * grand_z_Flexibility_2022_MINKOV +

group_z_Size_of_town_clean * grand_z_Individualism2022_MINKOV +

group_z_Size_of_town_clean * grand_z_Flexibility_2022_MINKOV +

group_z_Education_clean * grand_z_Individualism2022_MINKOV +

group_z_Education_clean * grand_z_Flexibility_2022_MINKOV +

group_z_Income_clean * grand_z_Individualism2022_MINKOV +

group_z_Income_clean * grand_z_Flexibility_2022_MINKOV +

Unemployed01 * grand_z_Individualism2022_MINKOV +

Unemployed01 * grand_z_Flexibility_2022_MINKOV +

(1 | Country_live_factor), binomial(link = "logit"),

data = d)

lmtest::lrtest(glmer_model_all, glmer_model_all_interactions, glmer_model_all_interactions_slope)

##################################### PLOTS

glmer_model_all_interactions_slope_raw <- lme4::glmer(Single01 ~ 1 +

Age + Sex01 + Size_of_town_clean + Education_clean + Income_clean + Unemployed01 +

Individualism2022_MINKOV + Flexibility_2022_MINKOV +

Age * Individualism2022_MINKOV +

Age * Flexibility_2022_MINKOV +

Sex01 * Individualism2022_MINKOV +

Sex01 * Flexibility_2022_MINKOV +

Size_of_town_clean * Individualism2022_MINKOV +

Size_of_town_clean * Flexibility_2022_MINKOV +

Education_clean * Individualism2022_MINKOV +

Education_clean * Flexibility_2022_MINKOV +

Income_clean * Individualism2022_MINKOV +

Income_clean * Flexibility_2022_MINKOV +

Unemployed01 * Individualism2022_MINKOV +

Unemployed01 * Flexibility_2022_MINKOV +

(1 + Age + Sex01 + Size_of_town_clean + Education_clean + Income_clean + Unemployed01| Country_live_factor),

binomial(link = "logit"),

data = d)

#############################################################

#############################################################

#############################################################

######################## Age × Individualism

effects_model2 <- effects::effect(term="Age * Individualism2022_MINKOV", mod=glmer_model_all_interactions_slope_raw,

xlevels=list(Age=c(mean(d$Age, na.rm=TRUE) - sd(d$Age, na.rm=TRUE), mean(d$Age, na.rm=TRUE) + sd(d$Age, na.rm=TRUE)),

Individualism2022_MINKOV=c(mean(d$Individualism2022_MINKOV, na.rm=TRUE) - sd(d$Individualism2022_MINKOV, na.rm=TRUE),

mean(d$Individualism2022_MINKOV, na.rm=TRUE) + sd(d$Individualism2022_MINKOV, na.rm=TRUE))))

effectsdata <- as.data.frame(effects_model2)

effectsdata <- effectsdata %>%

dplyr::mutate(Individualism2022_MINKOV_factor = factor(Individualism2022_MINKOV,

levels = c(mean(d$Individualism2022_MINKOV, na.rm=TRUE) - sd(d$Individualism2022_MINKOV, na.rm=TRUE),

mean(d$Individualism2022_MINKOV, na.rm=TRUE) + sd(d$Individualism2022_MINKOV, na.rm=TRUE)),

labels = c("Lower (-1SD)", "Higher (+1SD)")))

ggplot(data=effectsdata, aes(x=Age, y=fit, color = Individualism2022_MINKOV_factor, group=Individualism2022_MINKOV_factor)) +

geom_point() +

geom_line() +

geom_errorbar(aes(ymin=lower, ymax=upper), width=.15) +

xlab("Age") +

ylab("Likelihood of being a single") +

ggtitle("Likelihood of being a single \nacross participants' age and country-level individualism") + theme_bw() +

theme(axis.title=element_text(size=16),

axis.text=element_text(size=14),

legend.text=element_text(size=14),

legend.title=element_text(size=14),

plot.title=element_text(size=16, hjust=.5)) + scale_color_manual("Country-level individualism", values=c("salmon","turquoise"))

######################## Age × Flexibility_2022_MINKOV

effects_model2 <- effects::effect(term="Age * Flexibility_2022_MINKOV", mod=glmer_model_all_interactions_slope_raw,

xlevels=list(Age=c(mean(d$Age, na.rm=TRUE) - sd(d$Age, na.rm=TRUE), mean(d$Age, na.rm=TRUE) + sd(d$Age, na.rm=TRUE)),

Flexibility_2022_MINKOV=c(mean(d$Flexibility_2022_MINKOV, na.rm=TRUE) - sd(d$Flexibility_2022_MINKOV, na.rm=TRUE),

mean(d$Flexibility_2022_MINKOV, na.rm=TRUE) + sd(d$Flexibility_2022_MINKOV, na.rm=TRUE))))

effectsdata <- as.data.frame(effects_model2)

effectsdata <- effectsdata %>%

dplyr::mutate(Flexibility_2022_MINKOV_factor = factor(Flexibility_2022_MINKOV,

levels = c(mean(d$Flexibility_2022_MINKOV, na.rm=TRUE) - sd(d$Flexibility_2022_MINKOV, na.rm=TRUE),

mean(d$Flexibility_2022_MINKOV, na.rm=TRUE) + sd(d$Flexibility_2022_MINKOV, na.rm=TRUE)),

labels = c("Lower (-1SD)", "Higher (+1SD)")))

ggplot(data=effectsdata, aes(x=Age, y=fit, color = Flexibility_2022_MINKOV_factor, group=Flexibility_2022_MINKOV_factor)) +

geom_point() +

geom_line() +

geom_errorbar(aes(ymin=lower, ymax=upper), width=.15) +

xlab("Age") +

ylab("Likelihood of being a single") +

ggtitle("Likelihood of being a single \nacross participants' age and country-level flexibility") + theme_bw() +

theme(axis.title=element_text(size=16),

axis.text=element_text(size=14),

legend.text=element_text(size=14),

legend.title=element_text(size=14),

plot.title=element_text(size=16, hjust=.5)) + scale_color_manual("Country-level flexibility", values=c("salmon","turquoise"))

#############################################################

#############################################################

#############################################################

#############################################################

#############################################################

#############################################################

######################## Size_of_town_clean × Individualism

effects_model2 <- effects::effect(term="Size_of_town_clean * Individualism2022_MINKOV", mod=glmer_model_all_interactions_slope_raw,

xlevels=list(Size_of_town_clean=c(mean(d$Size_of_town_clean, na.rm=TRUE) - sd(d$Size_of_town_clean, na.rm=TRUE), mean(d$Size_of_town_clean, na.rm=TRUE) + sd(d$Size_of_town_clean, na.rm=TRUE)),

Individualism2022_MINKOV=c(mean(d$Individualism2022_MINKOV, na.rm=TRUE) - sd(d$Individualism2022_MINKOV, na.rm=TRUE),

mean(d$Individualism2022_MINKOV, na.rm=TRUE) + sd(d$Individualism2022_MINKOV, na.rm=TRUE))))

effectsdata <- as.data.frame(effects_model2)

effectsdata <- effectsdata %>%

dplyr::mutate(Individualism2022_MINKOV_factor = factor(Individualism2022_MINKOV,

levels = c(mean(d$Individualism2022_MINKOV, na.rm=TRUE) - sd(d$Individualism2022_MINKOV, na.rm=TRUE),

mean(d$Individualism2022_MINKOV, na.rm=TRUE) + sd(d$Individualism2022_MINKOV, na.rm=TRUE)),

labels = c("Lower (-1SD)", "Higher (+1SD)")))

ggplot(data=effectsdata, aes(x=Size_of_town_clean, y=fit, color = Individualism2022_MINKOV_factor, group=Individualism2022_MINKOV_factor)) +

geom_point() +

geom_line() +

geom_errorbar(aes(ymin=lower, ymax=upper), width=.15) +

xlab("Size of participants' town") +

ylab("Likelihood of being a single") +

ggtitle("Likelihood of being a single \nacross size of participants' towns and country-level individualism") + theme_bw() +

theme(axis.title=element_text(size=16),

axis.text=element_text(size=14),

legend.text=element_text(size=14),

legend.title=element_text(size=14),

plot.title=element_text(size=16, hjust=.4)) + scale_color_manual("Country-level individualism", values=c("salmon","turquoise"))

######################## Size_of_town_clean × Flexibility_2022_MINKOV

effects_model2 <- effects::effect(term="Size_of_town_clean * Flexibility_2022_MINKOV", mod=glmer_model_all_interactions_slope_raw,

xlevels=list(Size_of_town_clean=c(mean(d$Size_of_town_clean, na.rm=TRUE) - sd(d$Size_of_town_clean, na.rm=TRUE), mean(d$Size_of_town_clean, na.rm=TRUE) + sd(d$Size_of_town_clean, na.rm=TRUE)),

Flexibility_2022_MINKOV=c(mean(d$Flexibility_2022_MINKOV, na.rm=TRUE) - sd(d$Flexibility_2022_MINKOV, na.rm=TRUE),

mean(d$Flexibility_2022_MINKOV, na.rm=TRUE) + sd(d$Flexibility_2022_MINKOV, na.rm=TRUE))))

effectsdata <- as.data.frame(effects_model2)

effectsdata <- effectsdata %>%

dplyr::mutate(Flexibility_2022_MINKOV_factor = factor(Flexibility_2022_MINKOV,

levels = c(mean(d$Flexibility_2022_MINKOV, na.rm=TRUE) - sd(d$Flexibility_2022_MINKOV, na.rm=TRUE),

mean(d$Flexibility_2022_MINKOV, na.rm=TRUE) + sd(d$Flexibility_2022_MINKOV, na.rm=TRUE)),

labels = c("Lower (-1SD)", "Higher (+1SD)")))

ggplot(data=effectsdata, aes(x=Size_of_town_clean, y=fit, color = Flexibility_2022_MINKOV_factor, group=Flexibility_2022_MINKOV_factor)) +

geom_point() +

geom_line() +

geom_errorbar(aes(ymin=lower, ymax=upper), width=.15) +

xlab("Size of participants' town") +

ylab("Likelihood of being a single") +

ggtitle("Likelihood of being a single \nacross size of participants' town and country-level flexibility") + theme_bw() +

theme(axis.title=element_text(size=16),

axis.text=element_text(size=14),

legend.text=element_text(size=14),

legend.title=element_text(size=14),

plot.title=element_text(size=16, hjust=.5)) + scale_color_manual("Country-level flexibility", values=c("salmon","turquoise"))

#############################################################

#############################################################

#############################################################

#############################################################

#############################################################

#############################################################

######################## Education_clean × Individualism

effects_model2 <- effects::effect(term="Education_clean * Individualism2022_MINKOV", mod=glmer_model_all_interactions_slope_raw,

xlevels=list(Education_clean=c(mean(d$Education_clean, na.rm=TRUE) - sd(d$Education_clean, na.rm=TRUE), mean(d$Education_clean, na.rm=TRUE) + sd(d$Education_clean, na.rm=TRUE)),

Individualism2022_MINKOV=c(mean(d$Individualism2022_MINKOV, na.rm=TRUE) - sd(d$Individualism2022_MINKOV, na.rm=TRUE),

mean(d$Individualism2022_MINKOV, na.rm=TRUE) + sd(d$Individualism2022_MINKOV, na.rm=TRUE))))

effectsdata <- as.data.frame(effects_model2)

effectsdata <- effectsdata %>%

dplyr::mutate(Individualism2022_MINKOV_factor = factor(Individualism2022_MINKOV,

levels = c(mean(d$Individualism2022_MINKOV, na.rm=TRUE) - sd(d$Individualism2022_MINKOV, na.rm=TRUE),

mean(d$Individualism2022_MINKOV, na.rm=TRUE) + sd(d$Individualism2022_MINKOV, na.rm=TRUE)),

labels = c("Lower (-1SD)", "Higher (+1SD)")))

ggplot(data=effectsdata, aes(x=Education_clean, y=fit, color = Individualism2022_MINKOV_factor, group=Individualism2022_MINKOV_factor)) +

geom_point() +

geom_line() +

geom_errorbar(aes(ymin=lower, ymax=upper), width=.15) +

xlab("Participants' education") +

ylab("Likelihood of being a single") +

ggtitle("Likelihood of being a single \nacross participants' education and country-level individualism") + theme_bw() +

theme(axis.title=element_text(size=16),

axis.text=element_text(size=14),

legend.text=element_text(size=14),

legend.title=element_text(size=14),

plot.title=element_text(size=16, hjust=.5)) + scale_color_manual("Country-level individualism", values=c("salmon","turquoise"))

######################## Education_clean × Flexibility_2022_MINKOV

effects_model2 <- effects::effect(term="Education_clean * Flexibility_2022_MINKOV", mod=glmer_model_all_interactions_slope_raw,

xlevels=list(Education_clean=c(mean(d$Education_clean, na.rm=TRUE) - sd(d$Education_clean, na.rm=TRUE), mean(d$Education_clean, na.rm=TRUE) + sd(d$Education_clean, na.rm=TRUE)),

Flexibility_2022_MINKOV=c(mean(d$Flexibility_2022_MINKOV, na.rm=TRUE) - sd(d$Flexibility_2022_MINKOV, na.rm=TRUE),

mean(d$Flexibility_2022_MINKOV, na.rm=TRUE) + sd(d$Flexibility_2022_MINKOV, na.rm=TRUE))))

effectsdata <- as.data.frame(effects_model2)

effectsdata <- effectsdata %>%

dplyr::mutate(Flexibility_2022_MINKOV_factor = factor(Flexibility_2022_MINKOV,

levels = c(mean(d$Flexibility_2022_MINKOV, na.rm=TRUE) - sd(d$Flexibility_2022_MINKOV, na.rm=TRUE),

mean(d$Flexibility_2022_MINKOV, na.rm=TRUE) + sd(d$Flexibility_2022_MINKOV, na.rm=TRUE)),

labels = c("Lower (-1SD)", "Higher (+1SD)")))

ggplot(data=effectsdata, aes(x=Education_clean, y=fit, color = Flexibility_2022_MINKOV_factor, group=Flexibility_2022_MINKOV_factor)) +

geom_point() +

geom_line() +

geom_errorbar(aes(ymin=lower, ymax=upper), width=.15) +

xlab("Participants' education") +

ylab("Likelihood of being a single") +

ggtitle("Likelihood of being a single \nacross participants' education and country-level flexibility") + theme_bw() +

theme(axis.title=element_text(size=16),

axis.text=element_text(size=14),

legend.text=element_text(size=14),

legend.title=element_text(size=14),

plot.title=element_text(size=16, hjust=.5)) + scale_color_manual("Country-level flexibility", values=c("salmon","turquoise"))

#############################################################

#############################################################

#############################################################

#############################################################

#############################################################

#############################################################

######################## Income_clean × Individualism

effects_model2 <- effects::effect(term="Income_clean * Individualism2022_MINKOV", mod=glmer_model_all_interactions_slope_raw,

xlevels=list(Income_clean=c(1, 10),

Individualism2022_MINKOV=c(mean(d$Individualism2022_MINKOV, na.rm=TRUE) - sd(d$Individualism2022_MINKOV, na.rm=TRUE),

mean(d$Individualism2022_MINKOV, na.rm=TRUE) + sd(d$Individualism2022_MINKOV, na.rm=TRUE))))

effectsdata <- as.data.frame(effects_model2)

effectsdata <- effectsdata %>%

dplyr::mutate(Individualism2022_MINKOV_factor = factor(Individualism2022_MINKOV,

levels = c(mean(d$Individualism2022_MINKOV, na.rm=TRUE) - sd(d$Individualism2022_MINKOV, na.rm=TRUE),

mean(d$Individualism2022_MINKOV, na.rm=TRUE) + sd(d$Individualism2022_MINKOV, na.rm=TRUE)),

labels = c("Lower (-1SD)", "Higher (+1SD)")))

ggplot(data=effectsdata, aes(x=Income_clean, y=fit, color = Individualism2022_MINKOV_factor, group=Individualism2022_MINKOV_factor)) +

geom_point() +

geom_line() +

geom_errorbar(aes(ymin=lower, ymax=upper), width=.15) +

xlab("Participants' income") + xlim(1,10) +

ylab("Likelihood of being a single") +

ggtitle("Likelihood of being a single \nacross participants' income and country-level individualism") + theme_bw() +

theme(axis.title=element_text(size=16),

axis.text=element_text(size=14),

legend.text=element_text(size=14),

legend.title=element_text(size=14),

plot.title=element_text(size=16, hjust=.5)) + scale_color_manual("Country-level individualism", values=c("salmon","turquoise"))

######################## Income_clean × Flexibility_2022_MINKOV

effects_model2 <- effects::effect(term="Income_clean * Flexibility_2022_MINKOV", mod=glmer_model_all_interactions_slope_raw,

xlevels=list(Income_clean=c(mean(d$Income_clean, na.rm=TRUE) - sd(d$Income_clean, na.rm=TRUE), mean(d$Income_clean, na.rm=TRUE) + sd(d$Income_clean, na.rm=TRUE)),

Flexibility_2022_MINKOV=c(mean(d$Flexibility_2022_MINKOV, na.rm=TRUE) - sd(d$Flexibility_2022_MINKOV, na.rm=TRUE),

mean(d$Flexibility_2022_MINKOV, na.rm=TRUE) + sd(d$Flexibility_2022_MINKOV, na.rm=TRUE))))

effectsdata <- as.data.frame(effects_model2)

effectsdata <- effectsdata %>%

dplyr::mutate(Flexibility_2022_MINKOV_factor = factor(Flexibility_2022_MINKOV,

levels = c(mean(d$Flexibility_2022_MINKOV, na.rm=TRUE) - sd(d$Flexibility_2022_MINKOV, na.rm=TRUE),

mean(d$Flexibility_2022_MINKOV, na.rm=TRUE) + sd(d$Flexibility_2022_MINKOV, na.rm=TRUE)),

labels = c("Lower (-1SD)", "Higher (+1SD)")))

ggplot(data=effectsdata, aes(x=Income_clean, y=fit, color = Flexibility_2022_MINKOV_factor, group=Flexibility_2022_MINKOV_factor)) +

geom_point() +

geom_line() +

geom_errorbar(aes(ymin=lower, ymax=upper), width=.15) +

xlab("Participants' income") +

ylab("Likelihood of being a single") +

ggtitle("Likelihood of being a single \nacross participants' income and country-level flexibility") + theme_bw() +

theme(axis.title=element_text(size=16),

axis.text=element_text(size=14),

legend.text=element_text(size=14),

legend.title=element_text(size=14),

plot.title=element_text(size=16, hjust=.5)) + scale_color_manual("Country-level flexibility", values=c("salmon","turquoise"))

#############################################################

#############################################################

#############################################################

############# Sex Individualism

effects_model2 <- effects::effect(term="Sex01 * Individualism2022_MINKOV", mod=glmer_model_all_interactions_slope_raw,

xlevels=list(Sex01=c(0, 1),

Individualism2022_MINKOV=c(mean(d$Individualism2022_MINKOV, na.rm=TRUE) - sd(d$Individualism2022_MINKOV, na.rm=TRUE),

mean(d$Individualism2022_MINKOV, na.rm=TRUE) + sd(d$Individualism2022_MINKOV, na.rm=TRUE))))

effectsdata <- as.data.frame(effects_model2)

effectsdata <- effectsdata %>%

dplyr::mutate(Individualism2022_MINKOV_factor = factor(Individualism2022_MINKOV,

levels = c(mean(d$Individualism2022_MINKOV, na.rm=TRUE) - sd(d$Individualism2022_MINKOV, na.rm=TRUE),

mean(d$Individualism2022_MINKOV, na.rm=TRUE) + sd(d$Individualism2022_MINKOV, na.rm=TRUE)),

labels = c("Lower (-1SD)", "Higher (+1SD)")),

Sex01_factor = factor(Sex01, levels = c(0, 1), labels = c("Men", "Women")))

ggplot(data=effectsdata, aes(x=Sex01_factor, y=fit, color = Individualism2022_MINKOV_factor, group=Individualism2022_MINKOV_factor)) +

geom_point() +

geom_line() +

geom_errorbar(aes(ymin=lower, ymax=upper), width=.15) +

xlab("Sex") +

ylab("Likelihood of being a single") +

ggtitle("Likelihood of being a single \nacross participants' sex and country-level individualism") + theme_bw() +

theme(axis.title=element_text(size=16),

axis.text=element_text(size=14),

legend.text=element_text(size=14),

legend.title=element_text(size=14),

plot.title=element_text(size=16, hjust=.5)) + scale_color_manual("Country-level individualism", values=c("salmon","turquoise")) #+ xlim("0", "1")

#############################################################

#############################################################

#############################################################

############# Sex Flexibility_2022_MINKOV

effects_model2 <- effects::effect(term="Sex01 * Flexibility_2022_MINKOV", mod=glmer_model_all_interactions_slope_raw,

xlevels=list(Sex01=c(0, 1),

Flexibility_2022_MINKOV=c(mean(d$Flexibility_2022_MINKOV, na.rm=TRUE) - sd(d$Flexibility_2022_MINKOV, na.rm=TRUE),

mean(d$Flexibility_2022_MINKOV, na.rm=TRUE) + sd(d$Flexibility_2022_MINKOV, na.rm=TRUE))))

effectsdata <- as.data.frame(effects_model2)

effectsdata <- effectsdata %>%

dplyr::mutate(Flexibility_2022_MINKOV_factor = factor(Flexibility_2022_MINKOV,

levels = c(mean(d$Flexibility_2022_MINKOV, na.rm=TRUE) - sd(d$Flexibility_2022_MINKOV, na.rm=TRUE),

mean(d$Flexibility_2022_MINKOV, na.rm=TRUE) + sd(d$Flexibility_2022_MINKOV, na.rm=TRUE)),

labels = c("Lower (-1SD)", "Higher (+1SD)")),

Sex01_factor = factor(Sex01, levels = c(0, 1), labels = c("Men", "Women")))

ggplot(data=effectsdata, aes(x=Sex01_factor, y=fit, color = Flexibility_2022_MINKOV_factor, group=Flexibility_2022_MINKOV_factor)) +

geom_point() +

geom_line() +

geom_errorbar(aes(ymin=lower, ymax=upper), width=.15) +

xlab("Employment status") +

ylab("Likelihood of being a single") +

ggtitle("Likelihood of being a single \nacross participants' employment status and country-level flexibility") + theme_bw() +

theme(axis.title=element_text(size=16),

axis.text=element_text(size=14),

legend.text=element_text(size=14),

legend.title=element_text(size=14),

plot.title=element_text(size=16, hjust=.5)) + scale_color_manual("Country-level flexibility", values=c("salmon","turquoise")) #+ xlim("0", "1")

#############################################################

#############################################################

#############################################################

#############################################################

#############################################################

#############################################################

############# Unemployed01 Individualism

effects_model2 <- effects::effect(term="Unemployed01 * Individualism2022_MINKOV", mod=glmer_model_all_interactions_slope_raw,

xlevels=list(Unemployed01=c(0, 1),

Individualism2022_MINKOV=c(mean(d$Individualism2022_MINKOV, na.rm=TRUE) - sd(d$Individualism2022_MINKOV, na.rm=TRUE),

mean(d$Individualism2022_MINKOV, na.rm=TRUE) + sd(d$Individualism2022_MINKOV, na.rm=TRUE))))

effectsdata <- as.data.frame(effects_model2)

effectsdata <- effectsdata %>%

dplyr::mutate(Individualism2022_MINKOV_factor = factor(Individualism2022_MINKOV,

levels = c(mean(d$Individualism2022_MINKOV, na.rm=TRUE) - sd(d$Individualism2022_MINKOV, na.rm=TRUE),

mean(d$Individualism2022_MINKOV, na.rm=TRUE) + sd(d$Individualism2022_MINKOV, na.rm=TRUE)),

labels = c("Lower (-1SD)", "Higher (+1SD)")),

Sex01_factor = factor(Unemployed01, levels = c(0, 1), labels = c("Employed", "Unemployed")))

ggplot(data=effectsdata, aes(x=Sex01_factor, y=fit, color = Individualism2022_MINKOV_factor, group=Individualism2022_MINKOV_factor)) +

geom_point() +

geom_line() +

#geom_ribbon(aes(ymin=lower, ymax=upper), alpha=.3) +

geom_errorbar(aes(ymin=lower, ymax=upper), width=.15) +

xlab("Employment status") + #xlim(-2,2) +

ylab("Likelihood of being a single") + #ylim(1,7) +

ggtitle("Likelihood of being a single \nacross participants' employment status and country-level individualism") + theme_bw() +

theme(axis.title=element_text(size=16),

axis.text=element_text(size=14),

legend.text=element_text(size=14),

legend.title=element_text(size=14),

plot.title=element_text(size=16, hjust=.3)) + scale_color_manual("Country-level individualism", values=c("salmon","turquoise")) #+ xlim("0", "1")

###################################################################

############# Unemployed01 Flexibility_2022_MINKOV

effects_model2 <- effects::effect(term="Unemployed01 * Flexibility_2022_MINKOV", mod=glmer_model_all_interactions_slope_raw,

xlevels=list(Unemployed01=c(0, 1),

Flexibility_2022_MINKOV=c(mean(d$Flexibility_2022_MINKOV, na.rm=TRUE) - sd(d$Flexibility_2022_MINKOV, na.rm=TRUE),

mean(d$Flexibility_2022_MINKOV, na.rm=TRUE) + sd(d$Flexibility_2022_MINKOV, na.rm=TRUE))))

effectsdata <- as.data.frame(effects_model2)

effectsdata <- effectsdata %>%

dplyr::mutate(Flexibility_2022_MINKOV_factor = factor(Flexibility_2022_MINKOV,

levels = c(mean(d$Flexibility_2022_MINKOV, na.rm=TRUE) - sd(d$Flexibility_2022_MINKOV, na.rm=TRUE),

mean(d$Flexibility_2022_MINKOV, na.rm=TRUE) + sd(d$Flexibility_2022_MINKOV, na.rm=TRUE)),

labels = c("Lower (-1SD)", "Higher (+1SD)")),

Unemployed01_factor = factor(Unemployed01, levels = c(0, 1), labels = c("Employed", "Unemployed")))

ggplot(data=effectsdata, aes(x=Unemployed01_factor, y=fit, color = Flexibility_2022_MINKOV_factor, group=Flexibility_2022_MINKOV_factor)) +

geom_point() +

geom_line() +

geom_errorbar(aes(ymin=lower, ymax=upper), width=.15) +

xlab("Employment status") +

ylab("Likelihood of being a single") +

ggtitle("Likelihood of being a single \nacross participants' employment status and country-level flexibility") + theme_bw() +

theme(axis.title=element_text(size=16),

axis.text=element_text(size=14),

legend.text=element_text(size=14),

legend.title=element_text(size=14),

plot.title=element_text(size=16, hjust=.5)) + scale_color_manual("Country-level flexibility", values=c("salmon","turquoise")) #+ xlim("0", "1")

########## Table SM characteristics of the sample

kable(digits = 2,

d %>%

group_by(Country_live) %>%

summarise(

m = mean(Income_clean, na.rm=TRUE),

sd = sd(Income_clean, na.rm=TRUE),

min = min(Income_clean),

max = max(Income_clean))

) %>%

kable_styling(full_width = F)

kable(digits = 2,

d %>%

filter(!is.na(Unemployed01)) %>%

group_by(Country_live, Unemployed01) %>%

tally() %>%

mutate(freq = paste0(round(100 * n/sum(n), 1), "%")) %>%

arrange()) %>%

kable_styling(full_width = F)

summary(d$Single01)

sum(!is.na(d$Sex01))-sum(d$Sex01, na.rm=TRUE)

summary(d$Sex01)

mean(d$Age, na.rm=TRUE)

sd(d$Age, na.rm=TRUE)

mean(d$Size_of_town_clean, na.rm=TRUE)

sd(d$Size_of_town_clean, na.rm=TRUE)

mean(d$Education_clean, na.rm=TRUE)

sd(d$Education_clean, na.rm=TRUE)

mean(d$Size_of_town_clean, na.rm=TRUE)

sd(d$Size_of_town_clean, na.rm=TRUE)

mean(d$Income_clean, na.rm=TRUE)

sd(d$Income_clean, na.rm=TRUE)

sum(d$Unemployed01, na.rm=TRUE)

summary(d$Unemployed01)

# Revision 1 PLOS

# Additional analysis with those widowed/separated/divorced as 0

glmer_model_all_interactions_slope_PLOS <- lme4::glmer(Single01 ~ 1 +

group_z_Age + Sex01 + group_z_Size_of_town_clean + group_z_Education_clean + group_z_Income_clean + Unemployed01 +

grand_z_Individualism2022_MINKOV + grand_z_Flexibility_2022_MINKOV +

group_z_Age * grand_z_Individualism2022_MINKOV +

group_z_Age * grand_z_Flexibility_2022_MINKOV +

Sex01 * grand_z_Individualism2022_MINKOV +

Sex01 * grand_z_Flexibility_2022_MINKOV +

group_z_Size_of_town_clean * grand_z_Individualism2022_MINKOV +

group_z_Size_of_town_clean * grand_z_Flexibility_2022_MINKOV +

group_z_Education_clean * grand_z_Individualism2022_MINKOV +

group_z_Education_clean * grand_z_Flexibility_2022_MINKOV +

group_z_Income_clean * grand_z_Individualism2022_MINKOV +

group_z_Income_clean * grand_z_Flexibility_2022_MINKOV +

Unemployed01 * grand_z_Individualism2022_MINKOV +

Unemployed01 * grand_z_Flexibility_2022_MINKOV +

(1 + group_z_Age + Sex01 + Size_of_town_clean + Education_clean + Income_clean + Unemployed01| Country_live_factor),

binomial(link = "logit"),

data = additionalanalyses)

summary(glmer_model_all_interactions_slope_PLOS)

results_interactions_PLOS <- parameters::model_parameters(glmer_model_all_interactions_slope_PLOS, standardize = "refit", digits = 3, ci_digits = 3)

results_interactions_jtools_PLOS <- jtools::summ(glmer_model_all_interactions_slope_PLOS, digits = 3)

###### Compare the models with log likelihood logLik

lmtest::lrtest(glmer_model_all, glmer_model_all_interactions_slope_PLOS)

# VIFs

car::vif(glmer_model_all_interactions_slope_PLOS)

# Odds ratio

exp(lme4::fixef(glmer_model_all_interactions_slope_PLOS))

jtools::summ(glmer_model_all_interactions_slope_PLOS, digits = 3)

)

## Addition of r2 for individual and country variables of main model

# R2 of individual and country-level

############### Minkov

glmer_model_individual <- lme4::glmer(Single01 ~ 1 + group_z_Age + Sex01 + group_z_Size_of_town_clean + group_z_Education_clean + group_z_Income_clean + Unemployed01 +

(1 + group_z_Age + Sex01 + Size_of_town_clean + Education_clean + Income_clean + Unemployed01| Country_live_factor),

binomial(link = "logit"),

data = nomiss)

summary(glmer_model_individual)

results_interactions <- parameters::model_parameters(glmer_model_individual, standardize = "refit", digits = 3, ci_digits = 3)

results_interactions_jtools <- jtools::summ(glmer_model_individual, digits = 3)

############### Country

glmer_model_country <- lme4::glmer(Single01 ~ 1 + grand_z_Individualism2022_MINKOV + grand_z_Flexibility_2022_MINKOV +

(1 | Country_live_factor),

binomial(link = "logit"),

data = nomiss)

summary(glmer_model_country)

results_interactions <- parameters::model_parameters(glmer_model_country, standardize = "refit", digits = 3, ci_digits = 3)

results_interactions_jtools <- jtools::summ(glmer_model_country, digits = 3)
